# Fbxo22 inhibits metastasis in triple-negative breast cancer through ubiquitin modification of KDM5A and regulation of H3K4me3 demethylation

**DOI:** 10.1007/s10565-022-09754-w

**Published:** 2022-09-16

**Authors:** Siqiaozhi Li, Jinsong He, Xin Liao, Yixuan He, Rui Chen, Junhui Chen, Sean Hu, Jia Sun

**Affiliations:** 1 Shenzhen Toyon Biotechnology Co., Ltd, Shenzhen, 518057 People’s Republic of China; 2 Shenzhen Beike Biotechnology Research Institute, Shenzhen, 518057 People’s Republic of China; 3 Department of Breast Surgery, Shenzhen Hospital of Peking University, Shenzhen, 518057 People’s Republic of China; 4 Intervention and Cell Therapy Center, Shenzhen Hospital of Peking University, No. 1120, Lianhua Road, Shenzhen, 518057 Guangdong Province People’s Republic of China

**Keywords:** Triple-negative breast cancer, F-box protein 22, KDM5A, p16, H3K4me3, Ubiquitination, DNA damage, Metastasis

## Abstract

The importance of Fbxo22 in carcinogenesis has been highly documented. Here, we discussed downstream regulatory factors of Fbxo22 in TNBC. RNA-sequencing was conducted for identifying differentially expressed genes, followed by construction of a regulatory network. Expression patterns of Fbxo22/KDM5A in TNBC were determined by their correlation with the prognosis analyzed. Then, regulation mechanisms between Fbxo22 and KDM5A as well as between KDM5A and H3K4me3 were assayed. After silencing and overexpression experiments, the significance of Fbxo22 in repressing tumorigenesis in vitro and in vivo was explored. Fbxo22 was poorly expressed, while KDM5A was highly expressed in TNBC. Patients with elevated Fbxo22, decreased KDM5A, or higher p16 had long overall survival. Fbxo22 reduced the levels of KDM5A by ubiquitination. KDM5A promoted histone H3K4me3 demethylation to downregulate p16 expression. Fbxo22 reduced KDM5A expression to enhance p16, thus inducing DNA damage as well as reducing tumorigenesis and metastasis in TNBC. Our study validated FBXO22 as a tumor suppressor in TNBC through ubiquitination of KDM5A and regulation of p16.

**Supplementary Information:**

The online version contains supplementary material available at 10.1007/s10565-022-09754-w.

## Introduction

Triple-negative breast cancer (TNBC) is characterized by deficiency of progesterone and estrogen receptors as well as the loss of amplification of human epidermal growth factor receptor-2, the three typical biomarkers in BC (Denkert et al. 2017). As a heterogeneous disorder, TNBC presents a threat to patients with undesirable oncologic outcomes (Qattan 2020). Surgery and chemotherapy are the main treatment modalities for most TNBC patients,however, advanced breast cancer progresses rapidly after recurrence and has poorer overall survival compared with other breast cancer subtypes (Yin et al. 2020). Therefore, uncovering the mechanisms of TNBC and identifying effective biomarkers are necessitated for TNBC prevention and treatment.

Ubiquitination, a post-translational protein modification, has been found to control various physiological and pathological processes, including oncogenesis (Vucic et al. 2011). Fbxo22 has been documented to be lowly expressed in TNBC cells (Johmura et al. 2018). Moreover, patients who have high Fbxo22 expression exhibit a higher prognostic survival rate (Sun et al. 2018). Fbxo22 also exerts inhibitory function in breast cancer cell migration and invasion by ubiquitinating SNAIL levels (Sun et al. 2018). More importantly, the critical role of ubiquitin-dependent protein degradation in various cellular processes has been highlighted, such as cell cycle, transcriptional regulation, DNA damage repair, and apoptosis, and Fbxo22, as an ubiquitin ligase, can be implicated in modulation of the DNA damage repair process (Johmura et al. 2020). KDM5A (also called JARID1A or RBP2) shows correlation with diverse human cancers, including tumors of the breast and lung (Blair et al. 2011; Teng et al. 2013, Yang et al. 2019, 2021a, b]. Importantly, KDM5A are demonstrated to demethylase-dependently reduce tumor suppressor genes such as p27, p21, and p16 (Zeng et al. 2010). p16 is a cyclin-dependent kinases (CDK) inhibitor encoded by the CDKN2A gene and has been demonstrated to act as tumor suppressors (Zhao et al. 2016). Moreover, p16 is found to be linked with the prognosis of TNBC and may possess potential values in the prognostic prediction of TNBC (Zhang et al. 2014). From the above evidence, we tried to assay the correlation among Fbxo22, KDM5A, and p16 with TNBC progression for finding a novel regulatory mechanism for TNBC growth and metastasis.

## Materials and methods

### Animal experiments

Female NOD/SCID mice (4–5 weeks old, weighing 18–22 g, Hunan SJA Laboratory Animal Co., Ltd, Changsha, China) were housed for 1 week in specific pathogen-free environment under constant humidity (45–50%) and temperature (25–27 °C) with 12-h day/night cycle daily. The mice were fasted for 12 h before drug administration and were allowed to eat and drink freely at other time. Animal experimental operations were ratified by the animal ethics committee of Shenzhen Beike Biotechnology Research Institute.

MDA-MB-231 cells (5 × 106/0.2 mL) were injected in situ into the inguinal fat pad of mice (*n* = 8) to construct a mouse model of TNBC. The mice were sacrificed 6 weeks after injection, while the TNBC tissues were dissected and removed, and mouse normal (*n* = 8) breast tissues were taken as control. Tissues were preserved at − 80 °C for RNA-Seq.

In the subsequent animal experiments, mice were randomly treated with lentiviral empty vector, lentivirus overexpressing Fbxo22 (Flag-Fbxo22), and/or KDM5A lentivirus (HA-KDM5A), with 8 mice in each treatment. Lentiviral-infected MDA-MB-231 cells (5 × 10^6^/0.2 mL) were injected in situ into inguinal fat pads of mice. After 1 week, the tumor width and length of mice under different treatment were checked once a week, followed by calculation of tumor volume. After 7 weeks, the mice were killed and the tumor tissues were excised, weighed, and photographed with a camera.

For the lung metastasis model, cells (2 × 10^6^/0.2 mL) were injected into the tail vein of the mice. Mice were killed 6 weeks after injection and lung tissues were removed by autopsy and metastatic nodules were counted by microscopy. The presence of micrometastases in the lung tissues was examined by H&E staining.

### RNA-Seq

Mammary tissues from TNBC mice (*n* = 3) and normal mice (*n*= 3) were collected for RNA-Seq (Illumina HiSeq) at the UW Cystic Fibrosis Genomics Core Center (Miyazaki et al. 2019). Sequencing library was generated using the NEBNext® Ultra™ RNA Library Prep Kit for Illumina® (NEB, Beverly, MA). Quality control was then completed employing RNA-SeQC v1.1.8 and the HTSeq-counts v0.7.2 was applied for counting. The R language limma package was utilized to identify the differentially expressed genes between TNBC and controls with |logFoldChange|> 1 and*p*-value < 0.05 as cutoff values.

### Bioinformatics analysis

Correlation between genes and prognosis of TNBC patients was analyzed by ExSurv based on TCGA database breast cancer samples (with *p* < 0.05 as screening criteria). ROC curves were obtained by ROC Plotter based on TNBC patient prognostic data to evaluate key factors and their efficacy of prognosis in TNBC patients. Factors were considered to have good predictive performance with AUC > 0.5 and *p* < 0.05. The ubiquitination loci of the factors were obtained via Ubibrowser. The H3K4me3 methylation sites of the factors were obtained via UCSC browser. Pearson correlation coefficients were measured using the R package psych.

### Cell culture

The human breast epithelial cell line MCF-10A (CRL-10317); the luminal-type breast cancer cell lines MCF-7 (HTB-22), T47D (HTB-133), and ZR-75–1 (CRL-1500); and the TNBC cell lines MDA-MB-231 (CRM-HTB-26), MDA-MB-468 (HTB-132), Hs578T (HTB-126), and HEK293T (CRL-3216) were all procured from ATCC (Manassas, VA). MCF-10A, MCF-7, T47D, ZR-75–1, Hs578T, and HEK293T cells were cultured in Dulbecco’s modified Eagle’s medium appended to 10% FBS, 100 U/mL penicillin, and 100 μg/mL streptomycin in a 37 °C thermostatic incubator (BB15, Thermo, Madison, MA) with 5% CO_2_, while MDA-MB-231 and MDA-MB-468 cells were cultured in L-15 medium (Gibco) appended to 10% FBS in a 37 °C thermostatic incubator without CO_2_.

### Lentiviral infection

Lentiviral vectors overexpressing Fbxo22 and KDM5A were prepared as previously described (Sun et al. 2018). The lentiviral packaging vectors sh-Fbxo22 (5′-GGAATTGTAGTGACTCCAATG-3′) and sh-NC were procured from GenePharma (Shanghai, China), while the sh-p16 (5′-CCCTAAGCGCACATTCATGT-3′, TRCN0000010483) and sh-KDM5A (5′-CCAGACTTACAGGGACACTTA-3′, TRCN0000014629) were procured from Sigma-Aldrich (St.Louis, MO). The lentivirus titer was 10^9^ TU/mL.

### Cell viability, clone formation, cell migration, and invasion assays

Breast cancer cells were seeded in 96-well plates (8 × 10^3^cells per well) and cell viability was tested by CCK-8 (Sigma-Aldrich). For clone formation assay, cells were seeded into 6-well plates (2000 cells/well) evenly. The cells were cultured for a fortnight under normal growth conditions; the dispersed single cells may divide and proliferate and eventually form aggregated cell clusters, each of which was a “colony” (Kabakov and Gabai 2018). Cells were fixed in 4% paraformaldehyde and stained with 0.5% crystal violet staining solution (0.5% w/v, Solarbio, Beijing, China) for 15 min, followed by counting using an inverted microscope. Migration and invasion (with 50 μL of Matrigel, 354,234, BD Biosciences, San Diego, CA) assays were completed in differently treated breast cancer cells (Torres et al. 2019).

### Immunofluorescence staining

Cells were seeded into fluorescent culture dishes and incubated overnight to allow adhesion. The irradiation beam was used to locally induce DNA damage within the cells by irradiating the cells at 30 Gy at 4 Gy/min with a γ-ray emitter (cobalt 60), and the medium was renewed immediately after irradiation. Cells were then fixed in 4% formaldehyde for 10 min and then permeabilized with PBS containing 0.2% Triton X-100 for 3 min. Cells were blocked with 10% goat serum for 40 min and incubated with the primary antibodies of rabbit anti-γH2AX (1:50, AP0687, ABclonal, Boston, MA) overnight at 4 °C. Subsequently, cells were then incubated with fluorescent secondary antibodies of Cy3 Goat Anti-Rabbit IgG (H + L) (1:50, AS007, ABclonal) at ambient temperature for 2 h. The nuclei were stained with 4′,6-diamidino-2-phenylindole (10,236,276,001, Roche, Shanghai, China) and the slides were observed under a fluorescent microscope (XSP-BM22AY, Shanghai Optical Instrument Factory, Shanghai, China) for observation.

### Flow cytometry

For cell cycle analysis, cells were digested with trypsin, washed with pre-cooled PBS, resuspended in pre-chilled ethanol, and incubated overnight at − 20 °C. The following day, cells were stained with PI (Invitrogen, Eugene, OR) followed by cell cycle assessment employing a flow cytometer (FACS Calibur, BD Biosciences).

Cell apoptosis was detected using a membrane-linked protein V-FITC/PI double staining kit (70-AP101-100, MultiSciences, Hangzhou, China) employing a flow cytometry.

### Co-IP

To immunoprecipitate the exogenous proteins, the indicated plasmids (Flag-Fbxo22 and HA-KDM5A) were transfected into HEK293T cells. Cell lysis was carried out in IP lysis buffer (P0013, Beyotime, Shanghai, China) containing protease and phosphatase inhibitors. Cell lysis buffer was obtained by centrifugation at 12,000 g for 20 min at 4 °C. The cell lysis buffer containing 200 μg protein was then incubated with anti-flag antibody (1:50, F3165, Sigma-Aldrich) or anti-HA antibody (1:50, #3724, Cell Signaling, Hercules, CA) for 4 h at 4 °C.

To immunoprecipitate endogenous proteins, the same method as above-described was performed. Antibodies of lysates, KDM5A (1:100, ab70892, Abcam, UK), and IgG (1:50, #3900, Cell Signaling), at a concentration of 1 μg/mg, were added to the cell lysate and incubated overnight at 4 °C. The antibody-protein complexes were then captured with protein A/G Sepharose microbeads (Santa Cruz, CA). The complexes were then subjected to immunoblotting with mouse anti-Fbxo22 (1:1000, sc-100736, Santa Cruz) and mouse anti-KDM5A (1:1000, ab78322, Abcam).

### Ubiquitination assay

The indicated plasmids (Flag-Fbxo22, HA-KDM5A, and V5-Ubiquitin) were transfected into HEK293T cells. Prior to collection, cells were treated as previously described (Zhang et al. 2019). The obtained lysates were assayed and immunoprecipitated with protein A/G agarose (Sigma) pre-conjugated with the indicated antibody anti-HA (1:50, #3724, Cell Signaling). In addition, to prevent detection of ubiquitination of the E3 ligase itself and proteins associated with KDM5A, ubiquitination assays were also performed under denaturing conditions. Transfected cells were lysed in lysis buffer and incubated with SDS-PAGE sample buffer at 100 °C for 8 min, followed by incubation with HA antibody for IP and Immunoblotting. Antibodies used for immunoblotting were anti-flag (1:50, F3165, Sigma), anti-HA (1:50, #3724, Cell Signaling), and anti-V5 (1:50, 13,202, Cell Signaling).

Additionally, the ubiquitination level of KDM5A in TNBC cell lines was also examined. The obtained lysates were immunoprecipitated with protein A/G agarose (Sigma) pre-conjugated with the antibody of anti-KDM5A (1:100, ab70892, Abcam) or anti-IgG (1:50, #3900, Cell Signaling). The expression of the relevant proteins was then detected by immunoblotting using the antibodies of mouse anti-KDM5A (1:1000, ab78322, Abcam) and anti-ubiquitin (1:1000, 04–263, Millipore).

### Protein half-life detection

TNBC cells were treated with cycloheximide (CHX, Sigma; 10 μM) for different times (0, 0.5, 1, and 2 h) to block protein synthesis. Each group of proteins was then extracted and protein levels of KDM5A were assessed using immunoblotting.

### ChIP

ChIP was implemented employing the diluted sonicated lysates. Incubation of cells with the antibodies of KDM5A (1:100, ab70892, Abcam, UK), H3K4me3 (A2357, 1:50, ABclonal), and IgG (#3900, 1:50, Cell Signaling) overnight at 4 °C was carried out, with IgG antibody as a NC. The recovered and purified DNA fragments were used as amplification templates. Immunoprecipitated p16 was tested by RT-qPCR using iQ SYBR Green Supermix (BioRad, Hercules, CA), in which p16 gene promoter primer sequences were designed and provided by Sangon Biotech (Shanghai, China).

### RT-qPCR

Trizol-extracted total RNA reagent (15,596,026, Invitrogen) was reversely transcribed to cDNA employing a PrimeScript RT reagent Kit (RR047A, Takara, Japan). Fast SYBR Green PCR kit (Applied Biosystems, Waltham, MA) and ABI PRISM 7500 RT-PCR system (Applied Biosystems) were utilized for RT-qPCR. The relative gene expression was assayed utilizing the 2^−ΔΔCt^ method and normalized to GAPDH. The primer design is summarized in Table S1.

### Immunoblotting

The protein extracts from breast cancer tissues and cells were electro-separated and transferred to PVDF membrane which was then incubated with primary antibodies of mouse anti-KDM5A (1:1000, ab78322, Abcam, UK), rabbit anti-p16 (1:1000, A0262, ABclonal), mouse anti-Fbxo22 (1:1000, sc-100736, Santa Cruz), rabbit anti-E-cadherin (1:1000, #3195, Cell Signaling), rabbit anti-N cadherin (1:1000, #13,116, Cell Signaling), rabbit anti-Vimentin (1:1000, #5741, Cell Signaling), rabbit anti-H3K4me3 (A2357, 1:1000, ABclonal), and rabbit anti-GAPDH (1:1000, #2118, Cell Signaling, internal reference) overnight at 4 °C. HRP-labeled goat anti-mouse (1:10,000, BA1050, Boster, Wuhan, China) or goat anti-rabbit IgG (1:10,000, BA1054, Boster) secondary antibodies were incubated with the membrane for 1 h at ambient temperature. The films were exposed in an Amersham Imager 600 (UK). Gray-scale analysis was then performed using ImageJ.

### H&E staining

Paraffin-embedded Sects. (5 μm thickness) were first conventionally dewaxed and hydrated by gradient alcohol, and then stained with hematoxylin solution (Solarbio) for 2 min, followed by eosin solution staining for 1 min. Observation of histomorphological changes were completed under a light microscope (XP-330, ShBingyu, Shanghai, China).

### Immunohistochemical staining

The protein level of KDM5A, p16, and Fbxo22 was detected using streptavidin peroxidase labeled by immunohistochemical peroxidase. Paraffin-embedded specimens of breast cancer tissues were serially sectioned (5 μm thickness) and immune-stained with primary antibodies of rabbit anti-KDM5A (1:1000, ab78322, Abcam, UK), rabbit anti-p16 (1:100, A0262, ABclonal), and rabbit anti-Fbxo22 (13,606–1-AP, Protein-tech, Wuhan, China) overnight at 4 °C. The samples were incubated at 37 °C for 20 min with biotin-labeled goat anti-rabbit secondary antibody (BA1003, Boster), followed by incubation of 50 μL of streptomyces anti-biotin–peroxidase solution for 10 min at ambient temperature. Color development was completed with 3,3′-diaminobenzidine and microscopic observation for the specimens was performed with IgG used as a NC. Protein-positive cells were identified by the brownish-yellow color of normal positive cells, and positive staining was statistically analyzed using ImageJ.

### Statistical analysis

All data were processed using SPSS 22.0 statistical software (IBM, USA) and graphPad Prism 8.0. Measurement data were described as mean ± standard deviation and unpaired *t*-tests were used for comparisons between two groups, while ANOVA for comparisons among multiple groups. The tumor volume at different time points was assayed by repeated measure ANOVA. *p* < 0.05 indicated as statistically significant.

## Results

### Low expression of Fbxo22 in TNBC tissues and cells

**Fig. 1:**
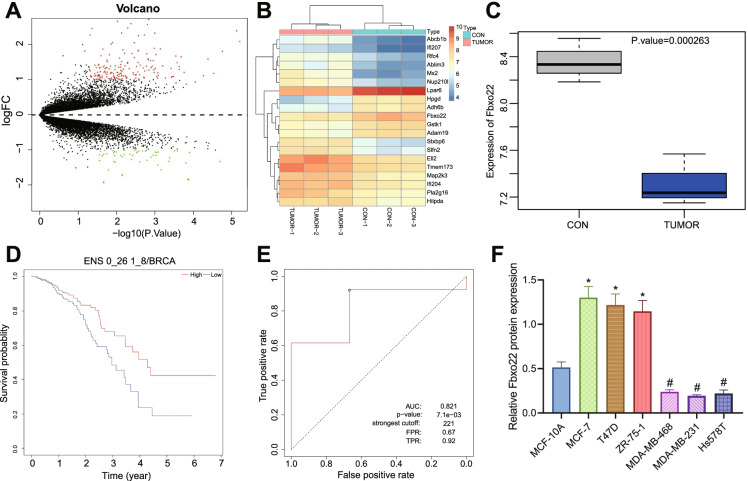
Bioinformatics analysis of Fbxo22 expression in TNBC tissues and their relationship with patient prognosis. **A** Results of differential analysis of RNA-seq sequencing data (volcano plot). The *X*-axis indicates -log10 (*p*-value) while the *Y*-axis indicates log2FC; red dots indicate highly expressed genes while green dots indicate low expressed genes. **B** Heat map plotted for the top 20 significantly differential genes with the smallest *p*-value. Color scale blue to red indicates gene expression values from small to large. **C** Differential expression of Fbxo22 in mouse TNBC tissues (*n* = 6) and normal breast tissues (*n* = 6). **D** Survival curve of Fbxo22 in breast cancer patients (high: 520, low: 521; hazard ratio = 1.557, *p*-value = 0.024). **E** ROC curve of Fbxo22 regarding prognosis of TNBC patients (*n* = 126). **F** Immunoblotting of Fbxo22 protein level in human normal breast epithelial cells, luminal type, and TNBC cells. * indicates *p* < 0*.*05 compared with MCF-10A cell. # indicates *p* < 0*.*05 compared with MCF-7 cell. Measurement data were expressed as mean ± standard deviation and one-way analysis of variance (ANOVA) used for comparisons among multiple groups. Cell experiments were repeated three times

By performing differential analysis of RNAseq in TNBC models and controls, we obtained 217 differentially expressed genes (Fig. 1A). The 20 significantly differential genes with the smallest *p*-value were selected to plot the heat map (Fig. 1B). Our RNAseq differential analysis depicted that Fbxo22 was significantly poorly expressed in TNBC tissues (Fig. 1C). In addition, survival analysis based on ExSurv database revealed that breast cancer patients with high expression of Fbxo22 had a higher survival rate (hazard ratio = 1.557, *p*-value = 0.024) (Fig. 1D, Tables S2, S3). ROC curve assessment based on ROC Plotter database also showed that Fbxo22 had good efficacy in predicting prognosis of TNBC patients (Fig. 1E).

Immunoblotting results confirmed that Fbxo22 was highly expressed in luminal-type breast cancer cells MCF-7, T47D, and ZR-75–1 compared to MCF-10A cells. However, low expression of Fbxo22 was observed in TNBC cells MDA-MB-231, MDA-MB-468, and Hs578T compared to luminal type breast cancer cells (Fig. 1F).

### Fbxo22 promotes KDM5A protein degradation through ubiquitination

**Fig. 2:**
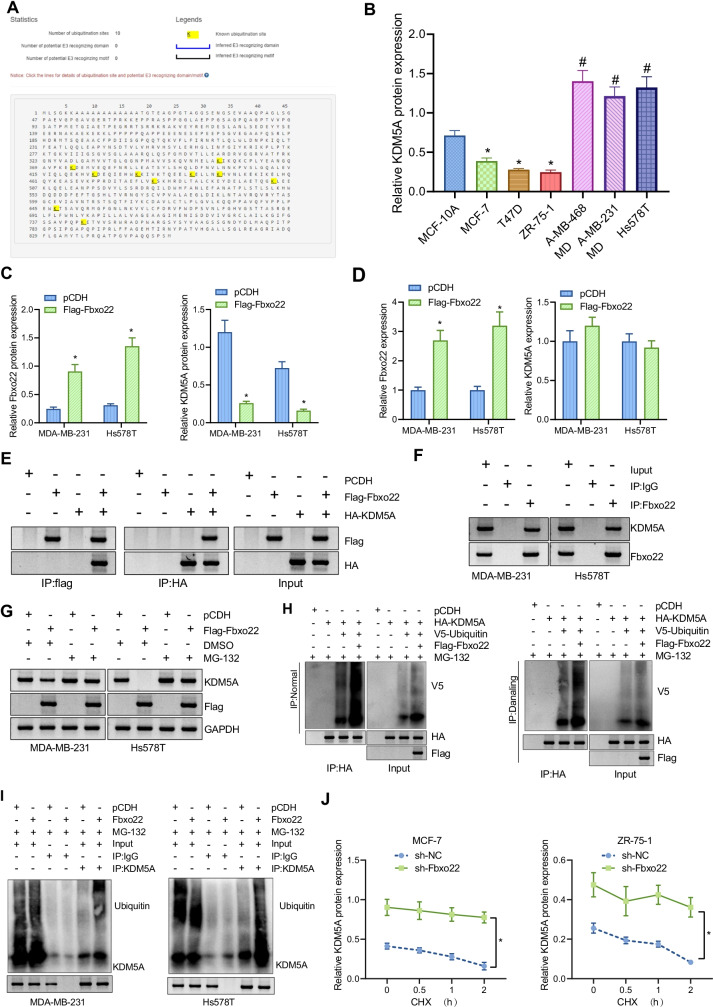
Fbxo22 regulates KDM5A levels through ubiquitination in TNBC cells. **A** E3 ubiquitination modification site of KDM5A. **B** Immunoblotting of KDM5A protein level in human normal breast epithelial cells, Luminal type, and TNBC cells. * indicates *p* < 0*.*05 compared with MCF-10A cell. # indicates *p* < 0*.*05 compared with MCF-7 cell. **C** Immunoblotting of protein levels of Fbxo22 and KDM5A in MDA-MB-231 and Hs578T cells after overexpression of Fbxo22. * indicates *p* < 0.05 compared with the pCDH group. **D** RT-qPCR detection of changes in mRNA expression of Fbxo22 and KDM5A in MDA-MB-231 and Hs578T cells after overexpression of Fbxo22. * indicates *p* < 0*.*05 compared with the pCDH group. **E** Co-IP assay to detect exogenous protein level of Fbxo22 and KDM5A. **F** Co-IP assay to detect the interaction between Fbxo22 and KDM5A at the endogenous protein level. **G** Immunoblotting of the protein levels of Fbxo22 and KDM5A in each group of MDA-MB-231 and Hs578T cells. **H** Co-IP assay to detect the ubiquitination of Fbxo22 on KDM5A at the endogenous protein level with MG-132 as a proteasome inhibitor, 5 μM for 6 h. **I** Co-IP assay to detect the ubiquitination of Fbxo22 on KDM5A at the endogenous protein level. **J** Immunoblotting of the effect of silencing Fbxo22 on CHX-induced KDM5A protein degradation; 10 μM, acting for 0, 0.5, 1, and 2 h, respectively. *p < 0.05. Measurement data were expressed as mean ± standard deviation and unpaired *t*-tests were used for comparisons between two groups, while one-way analysis of variance (ANOVA) or repeated measure ANOVA was used for comparisons among multiple groups. Cell experiments were repeated three times

By analysis of the Ubibrowser website, we further found that KDM5A had E3 ubiquitination modification sites (Fig. 2A). Therefore, we speculated that in breast cancer, Fbxo22 may degrade KDM5A protein by ubiquitination. Immunoblotting results confirmed that KDM5A was lowly expressed in luminal-type breast cancer cells MCF-7, T47D, and ZR-75–1 compared to MCF-10A cells. However, high expression of KDM5A was observed in TNBC cells MDA-MB-231, MDA-MB-468, and Hs578T compared to luminal type breast cancer cells (Fig. 2B).

To test the interaction between Fbxo22 and KDM5A, we overexpressed Fbxo22 in TNBC cells. The protein level of Fbxo22 was elevated in MDA-MB-231 and Hs578T cells treated with Flag-Fbxo22, while the protein level of KDM5A was diminished (Fig. 2C). Moreover, in MDA-MB-231 and Hs578T cells treated with Flag-Fbxo22, the mRNA expression of Fbxo22 was increased while the mRNA expression of KDM5A showed no alteration (Fig. 2D). The result indicates that Fbxo22 regulates KDM5A expression at the post-transcriptional level.

We then performed Co-IP experiments, which showed that Flag-Fbxo22 interacted with HA-KDM5A when co-expressed. Furthermore, by IP with anti-Fbxo22 or anti-KDM5A antibodies, interaction between Fbxo22 and KDM5A at the endogenous protein level was verified in MDA-MB-231 and Hs578T cells (Fig. 2E, F).

Whether Fbxo22 degrades KDM5A through ubiquitination was further investigated. Overexpression of Fbxo22 diminished the protein level of KDM5A, whereas treatment with MG-132 stabilized the KDM5A protein levels (Fig. 2G). Additionally, overexpression of Fbxo22 increased ubiquitination levels of KDM5A protein in cells under natural and denatured conditions, and overexpression of Fbxo22 in MDA-MB-231 and Hs578T cells enhanced the ubiquitination level of endogenous KDM5A (Fig. 2H, I).

In addition, the protein level of KDM5A continuously decreased with the prolonged action of CHX, while silencing Fbxo22 could inhibit the degradation of KDM5A protein and increase the protein half-life of KDM5A (Fig. 2J). Conclusively, Fbxo22 can reduce KDM5A levels in TNBC cells through ubiquitination.

### Fbxo22 inhibits malignant functions of TNBC cells through inhibition of KDM5A

**Fig. 3:**
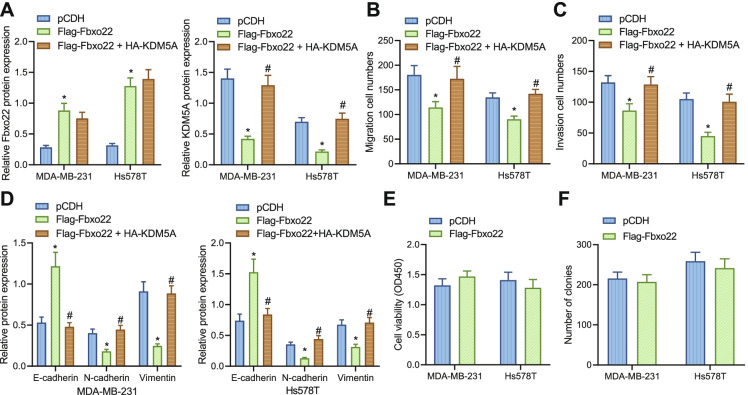
Fbxo22 represses the migration and invasion of TNBC cells by regulation of KDM5A. **A** Immunoblotting of protein levels of Fbxo22 and KDM5A in MDA-MB-231 and Hs578T cells after Flag-Fbxo22 or HA-KDM5A treatment. **B** Transwell assay to detect the migration ability of MDA-MB-231 and Hs578T cells after Flag-Fbxo22 or HA-KDM5A treatment. **C** Transwell assay of MDA-MB-231 and Hs578T cell invasion ability. **D** Immunoblotting of EMT-related protein levels in MDA-MB-231 and Hs578T cells after Flag-Fbxo22 or HA-KDM5A treatment. **E** CCK-8 was applied to detect cell viability. **F** Clone formation assay was used to detect cell clones. * indicates *p* < 0.05 compared with pCDH group; # indicates *p* < 0*.*05 compared with Flag-Fbxo22 group. Measurement data were expressed as mean ± standard deviation and one-way analysis of variance (ANOVA) was used for comparisons among multiple groups. Cell experiments were repeated three times

To further assay the mechanism by which Fbxo22 affects TNBC cell biological processes through the regulation of KDM5A, we overexpressed Fbxo22 and KDM5A in TNBC cells. The protein level of Fbxo22 was elevated following Flag-Fbxo22 treatment and that of KDM5A was diminished. In the cells treated with both Flag-Fbxo22 and HA-KDM5A, Fbxo22 protein level showed no alteration and KDM5A protein level was elevated (Fig. 3A).

The cell migration and invasion ability were curtailed after Flag-Fbxo22 treatment but enhanced after the co-treatment of Flag-Fbxo22 and HA-KDM5A (Fig. 3B, C). We also examined key proteins in the epithelial-mesenchymal transition (EMT) process, and E-cadherin protein level elevated while N-cadherin and Vimentin protein levels were diminished after Flag-Fbxo22 treatment. However, after the co-treatment of Flag-Fbxo22 and HA-KDM5A, the opposing trends appeared (Fig. 3D).

It has been documented that Fbxo22 can promote the proliferation of BC cells (Sun et al. 2018). Deletion of Fbxo22 itself does not affect the growth of T47D breast cancer tumors in female NOD/Scid mice and only slightly reduces the proliferation and clone abilities of MDA-MB 231 cells (Johmura et al. 2018; Bai et al. 2019). Therefore, we also tested the effect of overexpression of Fbxo22 on cell proliferation by CCK-8 and clone formation experiments and found that compared with the pCDH group, the Flag-Fbxo22 group had no significant changes in cell viability and clone formation (Fig.3E, F).

In conclusion, Fbxo22 can inhibit malignant phenotypes of TNBC cells by suppressing the level of KDM5A.

### KDM5A promotes histone H3K4me3 demethylation to downregulate p16 expression in TNBC cells

**Fig. 4:**
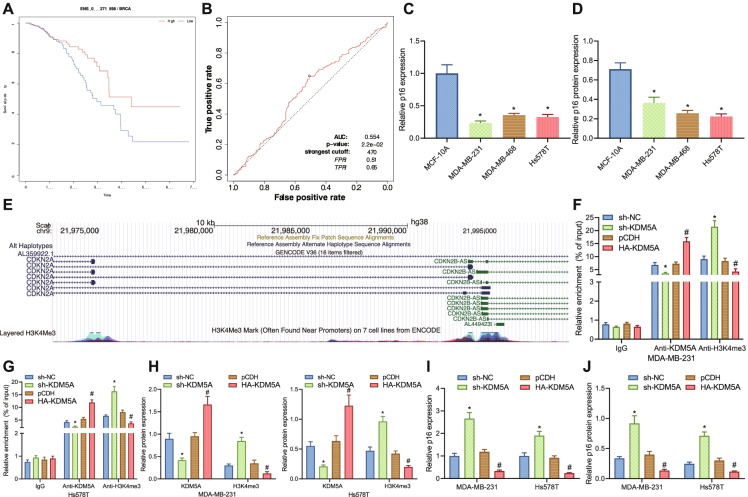
KDM5A regulation of histone H3K4me3 demethylation affects p16 gene expression in TNBC cells. **A** Survival curve of p16 in breast cancer patients (hazard ratio = 1.703, *p*-value = 0.008). **B** ROC curve of p16 on prognosis of TNBC patients. C, RT-qPCR of mRNA expression of p16 in human normal breast epithelial cells and TNBC cells. * indicates *p* < 0.05 compared with MCF-10A group. **D** Immunoblotting of p16 protein level in human normal breast epithelial cells and TNBC cells, * indicates *p* < 0.05 compared with MCF-10A group. **E** H3K4me3 methylation site map on p16 promoter obtained from UCSC browser website. **F**–**G** ChIP assay to detect the enrichment of KDM5A and H3K4me3 in the promoter region of p16 gene in MDA-MB-231 and Hs578T cells after sh-KDM5A or HA-KDM5A treatment, * indicates *p* < 0.05 compared with the sh-NC group; # indicates *p* < 0.05 compared to the pCDH group. **H** Immunoblotting of protein level of KDM5A and H3K4me3 in MDA-MB-231 and Hs578T cells after sh-KDM5A or HA-KDM5A treatment. * indicates *p* < 0.05 compared to the sh-NC group, # indicates *p* < 0.05 compared to the pCDH group. **I** RT-qPCR for mRNA expression of p16 in MDA-MB-231 and Hs578T cells after sh-KDM5A or HA-KDM5A treatment. * indicates *p* < 0.05 compared with sh-NC group, # indicates *p* < 0.05 compared with pCDH group. **J** Immunoblotting for protein level of p16 in MDA-MB-231 and Hs578T cells after sh-KDM5A or HA-KDM5A treatment, * indicates *p* < 0.05 compared with the sh-NC group, and # indicates *p* < 0.05 compared with pCDH group. Measurement data were expressed as mean ± standard deviation and one-way analysis of variance (ANOVA) was used for comparisons among multiple groups. Cell experiments were repeated 3 times

We also explored the relationship between p16 and survival of breast cancer patients by survival analysis and the results elaborated that breast cancer patients with high expression of p16 (alias: CDKN2A) had a higher survival rate (hazard ratio = 1.18, *p*-value = 0.0012) (Fig. 4A). Further assessment by ROC curves revealed that p16 had good efficacy in predicting the prognosis of patients with TNBC (Fig. 4B). We found that p16 was lowly expressed in MDA-MB-231 and Hs578T cells (Fig. 4C, D). These results unfold that p16 is lowly expressed in TNBC cells and shares correlation with prognostic survival of breast cancer patients.

Based on this, we further explored the mechanism of p16 involvement in the development of TNBC. The site prediction revealed a H3K4me3 site on the p16 promoter (Fig. 4E). Therefore, we hypothesized that KDM5A may downregulate p16 expression by promoting H3K4me3 demethylation of the p16 histone. Further ChIP depicted that in TNBC cells expressed silenced KDM5A, the enrichment of KDM5A in the p16 promoter region was greatly reduced, while the enrichment of H3K4me3 was elevated. While in the cells treated with HA-KDM5A, the enrichment of KDM5A in p16 promoter region increased, yet the enrichment of H3K4me3 was reduced (Fig. 4F, G). In addition, the treatment of sh-KDM5A decreased KDM5A protein level but increased H3K4me3 protein level in TNBC cells, while the treatment of HA-KDM5A brought about contrary findings (Fig. 4H). Silencing of KDM5A increased both mRNA and protein level of p16 in TNBC cells, while overexpression of KDM5A led to opposite trends (Fig. 4I, J).

The above results suggest that KDM5A promotes histone H3K4me3 demethylation, thereby downregulating p16 gene expression in TNBC cells.

### Fbxo22 induces DNA damage by promoting p16 expression in TNBC cells

**Fig. 5:**
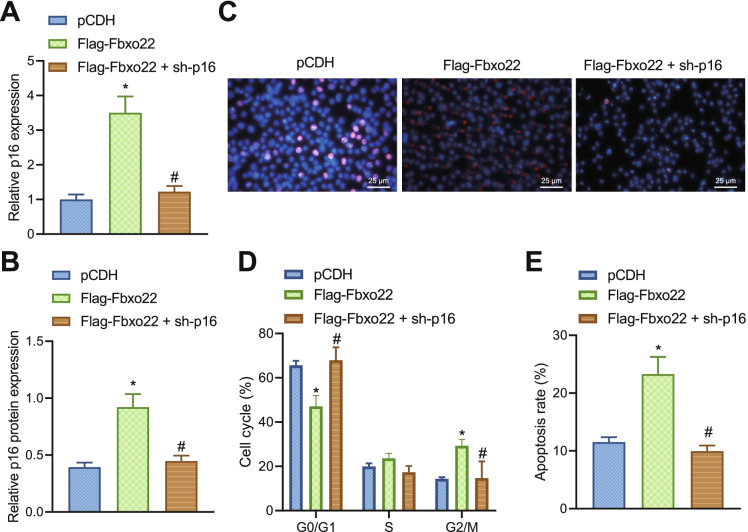
Fbxo22 affects DNA damage in TNBC cells by regulating p16 gene expression. **A** RT-qPCR detection of mRNA expression of p16 in MDA-MB-231 cells after Flag-Fbxo22 or sh-p16 treatment. **B** Immunoblotting of protein level of p16 in MDA-MB-231 cells after Flag-Fbxo22 or sh-p16 treatment. **C** Immunofluorescence staining detection of the enrichment of DNA damage marker γH2AX in the nucleus of each group after Flag-Fbxo22 or sh-p16 treatment (scale bar: 25 um). **D** Flow cytometry to detect the cell cycle in each group of cells after Flag-Fbxo22 or sh-p16 treatment. **E** Flow cytometry to detect apoptosis in each group of cells after Flag-Fbxo22 or sh-p16 treatment. * indicates *p* < 0.05 compared with pCDH group; # *p* < 0.05 compared with the Flag-Fbxo22 group. Measurement data were expressed as mean ± standard deviation and one-way analysis of variance (ANOVA) or repeated measure ANOVA was used for comparisons among multiple groups. Cell experiments were repeated 3 times

We further investigated whether Fbxo22 affects DNA damage in TNBC cells by regulating the expression of p16. The mRNA and protein level of p16 were increased in the cells treated with Flag-Fbxo22 but decreased after addition of sh-p16 (Fig. 5A, B). We then induced DNA damage by irradiation, and the immunofluorescence staining results depicted that overexpression of Fbxo22 enhanced the fluorescent signal of γH2AX in the nucleus and promoted DNA damage, while further silencing of p16 reduced the fluorescent signal of γH2AX in the nucleus and inhibited cellular DNA damage (Fig. 5C).

We further examined the cell cycle and apoptosis. There was a significant increase in G2/M phase cells after treatment of Flag-Fbxo22, while opposite trend was observed after addition of sh-p16 (Fig. 5D). Apoptosis was also enhanced in the cells treated with Flag-Fbxo22, while it was significantly decreased after the addition of sh-p16 (Fig. 5E). The above results suggest that Fbxo22 can promote DNA damage and apoptosis in TNBC cells by promoting p16 expression.

### Fbxo22 regulates KDM5A/p16 to inhibit metastasis in TNBC in vivo

**Fig. 6:**
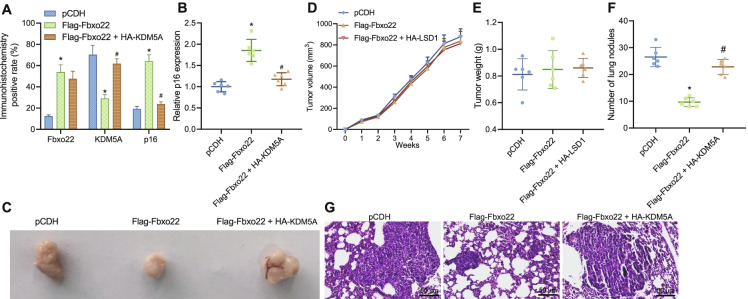
Effect of Fbxo22 regulation of KDM5A/p16 expression on tumourigenesis and metastasis of TNBC in vivo. **A** Immunohistochemical staining for protein level of Fbxo22, KDM5A, and p16 in breast cancer tissues of each group after Flag-Fbxo22 or HA-KDM5A treatment. **B** RT-qPCR for mRNA expression of p16 in breast cancer tissues of each group after Flag-Fbxo22 or HA-KDM5A treatment. **C** Anatomy of breast cancer tumors at week 7 in each group of mice after Flag-Fbxo22 or HA-KDM5A treatment. **D** Folding line of tumor volume growth from week 0 to week 7 in each group of mice after Flag-Fbxo22 or HA-KDM5A treatment. **E** Tumor weight statistics at week 7 in each group of mice after Flag-Fbxo22 or HA-KDM5A treatment. **F** Lung nodule count at week 6 of injection in each group of mice after Flag-Fbxo22 or HA-KDM5A treatment. **G** H&E staining to detect lung metastasis of breast cancer cells in each group of mice after Flag-Fbxo22 or HA-KDM5A treatment (*n* = 6) (scale bar: 50 um). * indicates *p* < 0.05 compared with pCDH group; ** indicates *p* < 0.01 compared with pCDH group; # indicates *p* < 0.05 compared with Flag-Fbxo22 group. Measurement data were expressed as mean ± standard deviation and one-way analysis of variance (ANOVA) or repeated measure ANOVA was used for comparisons among multiple groups

Finally, we investigated the effect of Fbxo22-regulated KDM5A/p16 expression on tumorigenesis and metastasis of TNBC in vivo by constructing a TNBC mouse model. As indicated by immunoblotting, the protein levels of Fbxo22 and p16 were elevated and that of KDM5A was decreased in TNBC tissues after treatment of Flag-Fbxo22. However, the expression of Fbxo22 in TNBC tissues was not significantly changed, while the protein level of KDM5A was increased and the protein level of p16 was diminished following both Flag-Fbxo22 and HA-KDM5A treatment (Fig. 6A). We obtained the same result trends in RT-qPCR as immunoblotting (Fig. 6B). Tumor volume and weight measurements showed that no significant alterations were seen in tumor volume and weight as compared with the Flag-Fbxo22 group with the pCDH group, and compared with the Flag-Fbxo22 group with the Flag-Fbxo22 + HA-KDM5A group (Fig. 6C–E).

We then constructed a metastatic tumor model and examined lung nodules after different treatments. There were fewer nodules in lung tissue after treatment of Flag-Fbxo22, while further addition of HA-KDM5A induced more nodules in lung tissues (Fig. 6F). After overexpression of Fbxo22, breast cancer metastases were reduced in the lung tissue, yet breast cancer metastases were more obvious after co-treatment of Flag-Fbxo22 and HA-KDM5A (Fig. 6G). The above results suggest that Fbxo22 can inhibit tumorigenesis and metastasis of TNBC in vivo by inhibiting KDM5A and promoting the expression of p16.

## Discussion

**Fig. 7:**
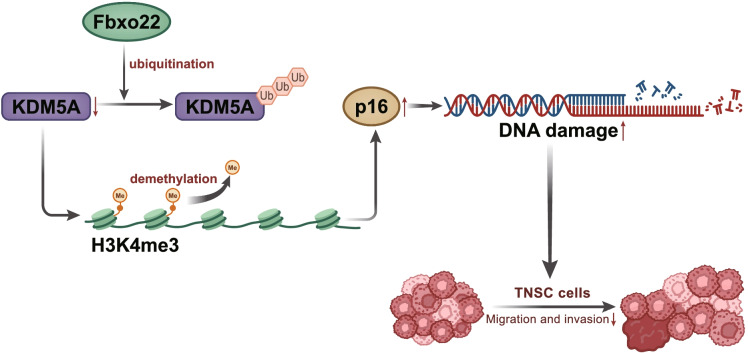
Schematic map of the regulatory role of Fbxo22 in TNBC with the involvement of KDM5A. Fbxo22 decreases KDM5A-mediated histone H3K4me3 demethylation through ubiquitination modification to increase p16 expression and DNA damage, thus suppressing metastasis of TNBC

Ubiquitin E3 ligases are capable of serving as oncoproteins or tumor suppressors in breast cancer (Xiao et al. 2016). Here, we probed into an F-box E3 ligase, Fbxo22 in TNBC. We highlighted the regulation of KDM5A by Fbxo22 through ubiquitin modification as well as the regulation of p16 by KDM5A via histone H3K4me3 demethylation in TNBC. Through cell and animal experiments, we made a conclusion that Fbxo22 could induce DNA damage through inhibiting the level of KDM5A and elevating p16 expression, thereby inhibiting the metastasis of TNBC (Fig.7).

As our work initially depicted, Fbxo22 was poorly expressed in TNBC cells and clinically collected tissues and patients with higher Fbxo22 showed higher survival rate. Similarly, a prior study showed that low Fbxo22 expression shared correlation with worse survival in human breast cancer, and knockdown of Fbxo22 promoted breast cancer cell invasiveness (Bai et al. 2019). Furthermore, Fbxo22 is an epigenetic multiplayer with its strikingly crucial role unveiled in cancer development and therapeutics (Johmura et al. 2020). Specifically, targeting Fbxo22 can be taken as a promising strategy for cancer therapy, since the inhibitory role of Fbxo22 has been proved in different malignancies, including breast cancer (Cheng et al. 2020). Moreover, we also found that overexpressed Fbxo22 by Flag-Fbxo22 was able to elevate E-cadherin but reduce N-cadherin and Vimentin. As previously described, EMT bears great responsibility in the invasive metastasis of breast cancer (Drasin et al. 2011). Consistently, one previous study also noted the inhibitory role of Fbxo22 in EMT in breast cancer (Sun et al. 2018).

Beside, our differential analysis of RNAseq showed that KDM5A was elevated in TNBC, and the low expression of KDM5A indicated higher survival rate among TNBC patients, which was consistent with the study reporting that high mRNA level of RBP2 was correlated with early and high incidence of tumor metastasis in breast cancer (Cao et al. 2014). We further explored the correlation between Fbxo22 and KDM5A, and the results displayed that Fbxo22 could promote the degradation of KDM5A protein level through ubiquitination. It has been reported that FBXO22 modulates changes in histone H3 marks and cognate transcriptional control pathways by regulation of KDM4A (Tan et al. 2011). However, the interaction between Fbxo22 and KDM5A remains for further investigation. In addition, KDM5A could downregulate p16 expression by promoting H3K4me3 demethylation. We found that higher p16 revealed higher survival rate. Similarly, p16 was proved to be an independent prognostic factor affecting the 5-year overall survival for TNBC (Zhang et al. 2014). Partly in line with our results, the cyclopenta[c]chromen derivative 1 (an identified antagonist of KDM5A) is capable of inducing production of p16 and p27 via blockage of KDM5A-mediated H3K4me3 demethylation, resulting in cell cycle arrest and senescence in breast cancer (Yang et al. 2019). Based on these existing works, we demonstrated that KDM5A could reduce p16 expression in TNBC through H3K4me3 demethylation. Moreover, TNBC shows the features of defects in DNA repair and thus sensitive to therapeutic concerning DNA-damaging (Telli et al. 2016). Low DNA repair capacity is linked with p16 mutations, being the risk factors for melanoma (Landi et al. 2005). However, how p16 affected DNA damage in TNBC is still unclear. At last, we explored the function of Fbxo22-KDM5A-p16 on tumoregenisis of TNBC in vivo. Since TNBC is highly aggressive and commonly develops lung metastasis (Wang et al. 2020), we observed lung nodules in xenograft tumors in mice. The animal experiments also illustrated that Fbxo22 could inhibit metastasis of TNBC in vivo by inhibiting KDM5A and promoting the expression of p16. The tumor-suppressing characteristics of Fbxo22 in breast cancer have been demonstrated before with its high expression prevented metastasis from the primary tumor site to lung (Bai et al. 2019).

In summary, we unveiled that Fbxo22 could induce DNA damage through decreasing KDM5A expression and elevating p16 expression, thus blocking the metastasis of TNBC. Specifically, Fbxo22 reduced KDM5A expression via ubiquitination, and KDM5A downregulated p16 expression by promoting histone H3K4me3 demethylation. Therefore, the identification of regulation of Fbxo22 on KDM5A-mediated p16 may function as a new modality for TNBC treatment in the future.

## Supplementary Information

Below is the link to the electronic supplementary material.

Supplementary file1 (DOC 31 KB)

Supplementary file2 (XLSX 58 KB)

Supplementary file3 (DOC 35 KB)

## Data Availability

The data that supports the findings of this study are available on request from the corresponding author.

## References

[CR1] Bai J, Wu K, Cao MH, Yang Y, Pan Y, Liu H (2019). SCF(FBXO22) targets HDM2 for degradation and modulates breast cancer cell invasion and metastasis. Proc Natl Acad Sci U S A.

[CR2] Blair LP, Cao J, Zou MR, Sayegh J, Yan Q (2011). Epigenetic regulation by lysine demethylase 5 (KDM5) enzymes in cancer. Cancers (Basel).

[CR3] Cao J, Liu Z, Cheung WK, Zhao M, Chen SY, Chan SW (2014). Histone demethylase RBP2 is critical for breast cancer progression and metastasis. Cell Rep.

[CR4] Cheng J, Lin M, Chu M, Gong L, Bi Y, Zhao Y (2020). Emerging role of FBXO22 in carcinogenesis. Cell Death Discov.

[CR5] Denkert C, Liedtke C, Tutt A, von Minckwitz G (2017). Molecular alterations in triple-negative breast cancer-the road to new treatment strategies. Lancet.

[CR6] Drasin DJ, Robin TP, Ford HL (2011). Breast cancer epithelial-to-mesenchymal transition: examining the functional consequences of plasticity. Breast Cancer Res.

[CR7] Johmura Y, Harris AS, Ohta T, Nakanishi M (2020). FBXO22, an epigenetic multiplayer coordinating senescence, hormone signaling, and metastasis. Cancer Sci.

[CR8] Johmura Y, Maeda I, Suzuki N, Wu W, Goda A, Morita M (2018). Fbxo22-mediated KDM4B degradation determines selective estrogen receptor modulator activity in breast cancer. J Clin Invest.

[CR9] Kabakov AE, Gabai VL (2018). Cell death and survival assays. Methods Mol Biol.

[CR10] Landi MT, Kanetsky PA, Tsang S, Gold B, Munroe D, Rebbeck T (2005). MC1R, ASIP, and DNA repair in sporadic and familial melanoma in a Mediterranean population. J Natl Cancer Inst.

[CR11] Miyazaki T, Gharib SA, Hsu YA, Xu K, Khodakivskyi P, Kobayashi A (2019). Cell-specific image-guided transcriptomics identifies complex injuries caused by ischemic acute kidney injury in mice. Commun Biol.

[CR12] Qattan A. Novel miRNA targets and therapies in the triple-negative breast cancer microenvironment: an emerging hope for a challenging disease. Int J Mol Sci. 2020;21(23). 10.3390/ijms21238905. 10.3390/ijms21238905 PMC7727826 33255471

[CR13] Sun R, Xie HY, Qian JX, Huang YN, Yang F, Zhang FL (2018). FBXO22 Possesses both protumorigenic and antimetastatic roles in breast cancer progression. Cancer Res.

[CR14] Tan MK, Lim HJ, Harper JW (2011). SCF(FBXO22) regulates histone H3 lysine 9 and 36 methylation levels by targeting histone demethylase KDM4A for ubiquitin-mediated proteasomal degradation. Mol Cell Biol.

[CR15] Telli ML, Timms KM, Reid J, Hennessy B, Mills GB, Jensen KC (2016). Homologous recombination deficiency (HRD) score predicts response to platinum-containing neoadjuvant chemotherapy in patients with triple-negative breast cancer. Clin Cancer Res.

[CR16] Teng YC, Lee CF, Li YS, Chen YR, Hsiao PW, Chan MY (2013). Histone demethylase RBP2 promotes lung tumorigenesis and cancer metastasis. Cancer Res.

[CR17] Torres A, Erices JI, Sanchez F, Ehrenfeld P, Turchi L, Virolle T (2019). Extracellular adenosine promotes cell migration/invasion of glioblastoma stem-like cells through A3 adenosine Receptor activation under hypoxia. Cancer Lett.

[CR18] Vucic D, Dixit VM, Wertz IE (2011). Ubiquitylation in apoptosis: a post-translational modification at the edge of life and death. Nat Rev Mol Cell Biol.

[CR19] Wang Z, Yang C, Li L, Jin X, Zhang Z, Zheng H (2020). Tumor-derived HMGB1 induces CD62L(dim) neutrophil polarization and promotes lung metastasis in triple-negative breast cancer. Oncogenesis.

[CR20] Xiao Z, Zhang P, Ma L (2016). The role of deubiquitinases in breast cancer. Cancer Metastasis Rev.

[CR21] Yang GJ, Ko CN, Zhong HJ, Leung CH, Ma DL. Structure-based discovery of a selective KDM5A inhibitor that exhibits anti-cancer activity via inducing cell cycle arrest and senescence in breast cancer cell lines. Cancers (Basel). 2019;11(1). 10.3390/cancers11010092. 10.3390/cancers11010092 PMC6360022 30650517

[CR22] Yang GJ, Wu J, Miao L, Zhu MH, Zhou QJ, Lu XJ, et al. Pharmacological inhibition of KDM5A for cancer treatment. Eur J Med Chem. 2021a;226:113855. 10.1016/j.ejmech.2021.113855. 10.1016/j.ejmech.2021.113855 34555614

[CR23] Yang GJ, Zhu MH, Lu XJ, Liu YJ, Lu JF, Leung CH (2021). The emerging role of KDM5A in human cancer. J Hematol Oncol.

[CR24] Yin L, Duan JJ, Bian XW, Yu SC (2020). Triple-negative breast cancer molecular subtyping and treatment progress. Breast Cancer Res.

[CR25] Zeng J, Ge Z, Wang L, Li Q, Wang N, Bjorkholm M (2010). The histone demethylase RBP2 Is overexpressed in gastric cancer and its inhibition triggers senescence of cancer cells. Gastroenterology.

[CR26] Zhang L, Chen J, Ning D, Liu Q, Wang C, Zhang Z (2019). FBXO22 promotes the development of hepatocellular carcinoma by regulating the ubiquitination and degradation of p21. J Exp Clin Cancer Res.

[CR27] Zhang S, Shao Y, Hou G, Bai J, Yuan W, Hu L (2014). QM-FISH analysis of the genes involved in the G1/S checkpoint signaling pathway in triple-negative breast cancer. Tumour Biol.

[CR28] Zhao R, Choi BY, Lee MH, Bode AM, Dong Z (2016). Implications of genetic and epigenetic alterations of CDKN2A (p16(INK4a)) in cancer. EBioMedicine.

