# Supplementary material for: Fbxo22 inhibits metastasis in triple-negative breast cancer through ubiquitin modification of KDM5A and regulation of H3K4me3 demethylation
Source: Cell Biol Toxicol. 2022 Sep 16;39(4):1641–55. doi: 10.1007/s10565-022-09754-w (PMC10425479; doi:10.1007/s10565-022-09754-w)
Supplement: Supplementary file 1 — Supplementary file1 (DOC 31 KB) [file 10565_2022_9754_MOESM1_ESM.doc]

**Table S1 RT-qPCR primer sequences**

| Gene | Primer sequences |
| --- | --- |
| Fbxo22 | F 5'-ATTGCTGTAAGGTGGGAGCC-3' |
|  | R 5'-ACCCCAAAGTGACAAAACCTG-3' |
| KDM5A | F 5'-GCCCTATTCCTGTGCGTCTT-3' |
|  | R 5'-TAAGAAACGTCCGCCCAGTC-3' |
| p16 | F 5'-CACCGAATAGTTACGGTCGG-3' |
|  | R 5'-GCACGGGTCGGGTGAGAGTG-3' |
| GAPDH | F 5'-CTCCTCCTGTTCGACAGTCAGC-3' |
|  | R 5'-CCCAATACGACCAAATCCGTT-3' |

Notes: Fbxo22, F-box protein 22; KDM5A, lysine demethylase 5A, GAPDH, glyceraldehyde 3-phosphate dehydrogenase; F, forward; R, reverse
