# Supplementary material for: Fbxo22 inhibits metastasis in triple-negative breast cancer through ubiquitin modification of KDM5A and regulation of H3K4me3 demethylation
Source: Cell Biol Toxicol. 2022 Sep 16;39(4):1641–55. doi: 10.1007/s10565-022-09754-w (PMC10425479; doi:10.1007/s10565-022-09754-w)
Supplement: Supplementary file 3 — Supplementary file3 (DOC 35 KB) [file 10565_2022_9754_MOESM3_ESM.doc]

# Table S3 Correlations of FBXO22 expression with clinical parameters of TNBC patients

| **Features** | | **High expression of FBXO22 (N [%])** | **Low expression of FBXO22 (N [%])** |  |
| --- | --- | --- | --- | --- |
| Age (years) | | | | |
| < 60 | 71 | 36 (50.70%) | 35 (49.30%) | 0.486 |
| ≥ 60 | 24 | 10 (41.67%) | 14 (58.33%) |
| TNM stage | | | | |
| I + II | 68 | 42 (61.76%) | 26 (38.24%) | < 0.001 |
| III | 27 | 4 (14.81%) | 23 (85.19%) |
| Tumor size | | | | |
| T1 + T2 | 64 | 36 (56.25%) | 28 (43.75%) | 0.031 |
| T3 + T4 | 31 | 10 (32.26%) | 21 (67.74%) |
| Lymph node metastasis | | | | |
| Negative | 32 | 22 (68.75%) | 10 (31.25%) | 0.009 |
| Positive | 63 | 24 (38.10%) | 39 (61.90%) |
